# Improving maternal and child nutrition in China: an analysis of nutrition policies and programs initiated during the 2000–2015 Millennium Development Goals era and implications for achieving the Sustainable Development Goals

**DOI:** 10.1186/s41043-020-00221-y

**Published:** 2020-12-02

**Authors:** Xin Huang, Bo Yang, Qin Liu, Ruilin Zhang, Shenglan Tang, Mary Story

**Affiliations:** 1 School of Public Health and Management, Research Center for Medicine and Social Development, Collaborative Innovation Center of Social Risks Governance in Health, Chongqing Medical University, No.1 Yixueyuan Road, Yuzhong District, Chongqing, 400016 P. R. China; 2 Information Center, The First Affiliated Hospital, Sun Yat-sen University, Guangzhou, 510080 P. R. China; 3 Department of Population Health Science and Duke Global Health Institute, Duke University, Durham, NC 27708 USA; 4 Duke Kunshan University, Kunshan, Jiangsu 215316 P. R. China; 5 Department of Family Medicine and Community Health and Duke Global Health Institute, Duke University, Durham, NC 27708 USA

**Keywords:** China, Nutrition, Maternal and child health, MDG, SDG

## Abstract

**Background:**

Although good progress was made in maternal and child nutrition during the Millennium Development Goals (MDGs) era, malnutrition remains one of the major threats on global health. Therefore, the United Nation set several nutrition-related goals in the Sustainable Development Goals (SDGs). There is much to be learned from individual countries in terms of efforts and actions taken to reduce malnutrition. China, as a developing country, launched a number of nutrition improvement policies and programs that resulted in dramatic progress in improving maternal and child nutrition during the MDGs era. This study explored the impact, experiences, and lessons learned from the nutrition policies and programs initiated in China during the MDGs era and implications to achieve the SDGs for China and other developing countries.

**Method:**

The CNKI database and official websites of Chinese government were searched for reviews on nutrition-related policies and intervention programs. A qualitative study was conducted among key informants from the Chinese government, non-governmental organizations (NGOs), and universities for two major national nutrition intervention programs.

**Results:**

The literature review documented that during the MDGs era, six nutrition policies and eight trans-province and nationwide nutrition intervention programs collectively made good progress in improving maternal and child nutrition in China. Nutrition policies tended to be targeted at infants and children, with less attention on reproductive and maternal nutrition. Nutrition intervention programs focused primarily on undernutrition and have achieved positive results, while for breastfeeding improvement and prevention and control on overweight and obesity were limited. Results from the qualitative study indicated that effective nutrition program implementation was facilitated through the cooperation of multiple sectors and by the government and NGO partnerships, however, still face challenges of insufficient operational funds from local governments and inadequacy of program monitoring and management.

**Conclusion:**

Nutrition policies and intervention programs promulgated in China during the MDGs era have made major contributions to the rapid decline of undernutrition and are in line to achieve the SDGs related to child wasting, stunting, low birth weight, and anemia in reproductive-age women. However, appropriate policies and program implementation are needed to improve exclusive breastfeeding rates and reduce obesity to achieve the SDGs in years to come.

**Supplementary Information:**

**Supplementary information** accompanies this paper at 10.1186/s41043-020-00221-y.

## Background

The Millennium Development Goals (MDGs), established by the United Nations (UN) in 2000, set out eight ambitious goals to be achieved by 2015. Two of them were directly linked to the health of children and women: (1) reduce by two thirds the mortality rate among children under five and (2) reduce by three quarters the maternal mortality ratio. During the MDGs era, maternal and child nutrition has been improved greatly in many developing countries through various intervention and policy strategies. The Global Nutrition Policy Review (2013) based on 119 World Health Organization (WHO) Member States indicated that most countries had a range of policies and regulations on nutrition [1], including breastfeeding and complementary feeding improvement strategies, micronutrient supplements for pregnant women, infants, and young children [2], school feeding programs [3], and child obesity interventions [4]. These interventions played an important role in improving child and maternal nutrition in many developing countries. Counseling or educational interventions increased exclusive breastfeeding (EBF) rates [5], supplementation with folate and iron reduced the risk of anemia and maternal mortality [6, 7], providing micronutrient supplementation to pregnant women reduced the risk of low birth weight [2], school feeding programs reduced the rate of stunting, wasting, and anemia [3], and school-based obesity interventions [4] and nutrition and agricultural policies [8] reduced the incidence of child obesity.

While good progress was made in maternal and child nutrition during the MDGs era, malnutrition remains one of the major threats on global health. According to the WHO, in 2016, an estimated 155 million children under the age of 5 were suffering from stunting, while 41 million were overweight or obese, roughly 45% of deaths among children under 5 years of age were linked to undernutrition [9]. Every country in the world is affected by malnutrition. WHO indicates [9] combating malnutrition in all its forms is one of the greatest global health challenges, diverse multi-sector interventions aimed at changing behaviors at the individual level combined with policies, systems and environmental changes need to be considered for implementation at a national scale to eliminate malnutrition. Therefore, the Sustainable Development Goals  (SDGs) set six specific goals for nutrition, including by 2025 (1) 40% reduction in children younger than 5 years who are stunted, (2) 50% reduction of anemia in women of reproductive age, (3) 30% reduction of low birth weight, (4) no increase in childhood overweight, (5) increase the rate of exclusive breastfeeding in the first 6 months up to at least 50%, and (6) reduce and maintain childhood wasting to < 5% [10, 11]. There is much to be learned from individual countries in terms of efforts and actions taken to reduce maternal and child malnutrition, in order to achieve the nutrition-related 2030 SGDs [10, 11].

China, as a developing country, launched a number of nutrition improvement policies and programs that resulted in dramatic progress and impact in improving maternal and child nutrition during the MDGs period from 2000 to 2015. It is reported that in China from 2002 to 2013 for children under 6 years, the rates of stunting were reduced in half from 16.3 to 8.1% and underweight from 5.7 to 2.5% [12]. Anemia rates for pregnant women decreased from 28.9 to 9.3% and among lactating mothers from 30.7 to 17.2% [12]. The mortality rate of children under 5 years of age dropped from 61.0 per 1000  in 1991 to 10.7 per 1000 in 2015 [13]. The national maternal mortality rate decreased from 88.8 per 100,000 in 1990 to 23.2 per 100,000 in 2013 [13].

Policy and program analysis enables us to better understand and strengthen the policy environment and the implementation of the intervention programs [14]. Interviews with stakeholders involved in developing and implementing nutrition intervention programs are important to better understand programs’ successes, challenges, and improvements needed in China to achieve the nutrition-related SDGs [10, 11].

For this study, the reviews of literature and policy documents evaluating nutrition-related policies and intervention programs in China during the MDGs (2000–2015) were conducted, and qualitative interviews were undertaken with key informants from the Chinese government, non-governmental organizations (NGOs), and universities for two major national nutrition programs. The aim of the study was to better understand the nutrition-related experiences and best practices in China, in order to achieve the 2030 nutrition-related SDGs [10, 11]. Further, experiences and lessons learned from China have implications for other developing countries facing similar nutrition and health problems that China faced two decades ago and could provide insights into improving maternal and child nutrition to achieve the nutrition-related SDGs.

## Methods

### Review of literature and policy documents

This study utilized the China National Knowledge Infrastructure (CNKI) database to search and collect the studies and documents on nutrition policies and interventions programs for Chinese women and children from January 2000 to December 2015. Studies were searched using the following terms: maternal nutrition, infant nutrition, child nutrition, nutrition policies, and intervention programs. We also checked references of included studies and documents in order to collect more information. Searches were also conducted of official websites of the Chinese government and related departments, such as Ministry of Education (MoE, http://www.moe.gov.cn) of China, National Health and Family Planning Commission of China (HFPC, http://www.nhc.gov.cn), and United Nations International Children’s Fund (UNICEF, https://www.unicef.org). In addition, grey documents were included in our study, and we also consulted with related experts and institutions for more detailed information about the nutrition policies and intervention programs for Chinese women and children.

### Qualitative interviews

Qualitative interviews with key informants knowledgeable on the maternal and child nutrition intervention programs were conducted at the national and provincial levels during June and July, 2017.

### Sampling and participants

Purposive sampling, as well as snowball sampling, was used to select the key informants. Consultants and Steering Committee members from the Gates-funded grant “Achieving Health-Related SDGs in China: Developing Evidence-based Options for Actions” (led by Duke Global Health Institute) helped with the selection of key informants. To better understand the implementation of nutrition policies and programs across the mainland of China, interviews with key stakeholders from both the national level and provincial level (Yunnan and Hubei) were conducted. China is a country with regional and the socioeconomic differences across provinces, which can be divided roughly into three regions: The Western part is a less developed region, the Middle is average, and the Eastern part is the most developed region. Yunnan province, located in Western China, was chosen to represent a less developed region and Hubei province (located in Middle China) to represent an average developed region. No province in the Eastern region was included since the nutrition intervention program was not be implemented in this region.

A total of 23 key informants participated in the interviews with 10 stakeholders from national sectors, six at the provincial level from Hubei, and seven from Yunnan. These informants had experience and expertise in maternal and child nutrition and included the chief leader from Chinese Centers for Disease Control and Prevention (CDCs), HFPC, MoE, maternal and child hospitals, All-China Women’s Federation (ACWF) and national foundations (e.g., UNICEF, China Children and Teenager’s Fund (CCTF), China Development Research Foundation (CDRF)), and nutrition intervention programs and experts from the top universities/institutions in China (see Table S1 for details).

### Interview methods

The interviews followed a semi-structured topic interview guide conducted in Chinese. The topic guide was formulated based on the literature reviews of intervention programs and consultation from nutrition experts, which was then adjusted according to the specialties of the interviewees, in order to obtain as much detailed information as possible. The interview guide mainly included three themes of each nutrition intervention program: (1) enabling factors, (2) challenges in program implementation, and (3) stakeholders’ views on sustainability. To ensure the authenticity and effectiveness of the interview, researchers contacted the high-level interviewees in advance, providing information on the context of the study and interview questions that would be asked during the interview.

The in-depth interviews were conducted in-person by two Chinese researchers (two of the co-authors). All interviews were recorded anonymously with informed consent. Each interview lasted about 45–60 min. This study was conducted according to the guidelines laid down in the Declaration of Helsinki, and all procedures involving research study participants were approved by the Ethics Committee of Duke University (2017-1359)

### Data analysis

#### Literature/policy document review and analysis

We systematically summarized for each article/report: the maternal and child nutrition-related policies (issued department, duration of implementation, target population, nutrition-related content) and intervention programs (target population, implementation area and duration, organizer, contents and effects) in China.

#### Qualitative data analysis

All the analysis stages were conducted independently by two researchers. A six-stage Framework Approach [15] was used to guide the qualitative analysis: (1) Transcription: all audio-taped interviews were transcribed verbatim by one researcher and kept anonymous and checked by another researcher. (2) Familiarization: all interviews were read in full text by 2 independent researchers simultaneously. Notes were made in the interview transcriptions if any important segments were identified. (3) Development of an analytical framework: thematic framework was developed based on the objectives, topic guide, and new themes from the interviewees. (4) Coding: MAXQDA (version 12) was used to manage transcriptions. Coding was performed by two researchers, one responsible for coding and the other responsible for checking. (5) Charting: a case display was formulated per interviewee based on thematic framework. Each case consisted of a short interview answer of each question. (6) Interpretation: summarize the main findings and present the results with key quotations in the text.

## Results

### Overview of nutrition intervention policies during the MDGs (2000–2015)

The results of the literature review documented that during the MDGs, the Chinese government enacted several national nutrition policies to improve maternal and child nutrition, and six policies showed the most impact (Table 1). Of those, three were issued by State Council, two by MoH, and one by MoE. Four of the six focused on children under 5 years of age and school-aged youth, one was aimed at women (the *Outline for the Development of Chinese woman*) and one (the *Outline for the Development of food and nutrition*) was for all Chinese residents, with emphasis on priority populations (e.g., infants and children, adolescents, women, and the elderly). For nutrition areas, two of the policies focused on undernutrition improvement of woman or school-age children without specific goals, the *Outline for the Development of Chinese Children, Infant feeding strategy* and *Technical specification and guidance on child feeding*, addressed the child undernutrition including low birth weight, stunting, anemia, micronutrient deficiency, and breastfeeding or complementary feeding; two of them had specific nutrition goals. The *Outline for the Development of Food and Nutrition in China* is the most comprehensive nutrition policy, which focused on improvement for undernutrition and infant feeding, but also took overweight and obesity into account. Priority was given to geographic regions (e.g., rural and western areas) and the food industry (e.g., dairy industry, soybean industry and food processing).

**Table 1:** Overview of nutrition intervention policies during the MDGs

Nutrition intervention policies	Issued department	Implemental duration (year)	Target population	Nutrition-related contents of the policy
Outline for the development of Chinese children [16, 17]	State Council	2001–2010	Children	Set five specific goals for child nutrition improvement: By 2010, • Moderate and severe malnutrition rates of children under 5 years: fell by 1/4 on the basis of 2000 • Low birth weight: less than 5% • Prevalence of infant parents’ knowledge of scientific feeding: more than 85% • Infants’ breastfeeding rate reaches 85% in provincial units, add supplementary foods timely and reasonably • Reduce child vitamin A deficiency
		2011–2020	Children	Set four goals specific goals for child nutrition improvement: By 2020, • Low birth weight: less than 4% • Exclusive breastfeeding in the first 6 months: at least 50% • Anemia of children under 5 years: less than 12% • Stunting and underweight of children under 5 years: less than 7% and 5% respectively
Outline for the development of Chinese woman [18]	State Council	2011–2020	Woman	Set one goal for woman nutrition improvement: • Reduce the prevalence of moderate and severe anemia in pregnant women
Outline for the development of food and nutrition in China [19, 20]	State Council	2001–2010	Chinese residents	Give priority to the development of • Three key food industries (dairy industry, soybean industry, and food processing industry) • Two key areas (rural and western areas) • Three key groups (adolescents, women and children, the elderly) Set three goals for improvement on maternal and child nutrition: By 2010, • Stunting and underweight of children under 5 years: less than 15% and 5% respectively • Anemia of pregnant woman and children under 5 years: less than 20% and 15% respectively • Promote the breastfeeding of infants within 4 months, and gradually supplement various supplementary foods for infants over 4 months old
		2014–2020	Chinese residents	Give priority to the development of • Three key products (high quality agricultural products, convenient nutrient processed foods, dairy and soy foods) • Three key areas (poor areas, rural areas, floating population gathering, and new urbanization areas) • Three key groups (maternal and infants, children and adolescents, the elderly) Set four goals for improvement on maternal and child nutrition: By 2020, • The stunting of children under 5 years: less than 7% • Anemia rate of the whole population: less than 10% • Anemia of pregnant and lactating woman, and children under 5 years: less than 17% and 12% respectively • Reduce the growth rate of overweight, obesity, and dyslipidemia of Chinese residents
Infant feeding strategy [21]	MoH	2007~	Infants	• Protect, promote, and support breastfeeding and increase the rate of exclusive breastfeeding in first 6 months • Add supplementary foods timely and reasonably • Increase breastfeeding rate of infants within 6 months • Prohibit selling formulate milk in hospitals • Strengthen public education on breastfeeding • Strengthen the standardized management of baby-friendly hospitals • Guide the rational addition of supplementary foods for infants over 6 months • Strengthen supervision of infant supplementary foods • Prevent child micronutrient deficiencies
Technical specification and guidance on child feeding and nutrition [22]	MoH	2012~	Children under 7 and their parents	Provide guidance for parents on breastfeeding, food conversion, reasonable diet, and dietary behavior.
Plan of the national medium- and long-term education reform and development (2010–2020) [23]	MoE	2010–2020	Chinese students (mainly)	Improve the nutritional status of students, in particular, launch the implementation of the nutritional improvement plan for rural primary and middle school students in minority and poverty-stricken areas.

*MoE* Ministry of Education, *MoH* Ministry of Health

### Overview of the nutrition intervention programs during the MDGs era

Findings from the literature review indicated that during the MDGs period, eight trans-province and national maternal and (or) child nutrition intervention programs were implemented. Most of these programs were organized by multiple government sectors and NGOs such as ACWF and UNICEF. Six of the programs aimed to improve child undernutrition, including wasting, stunting, underweight, micronutrient deficiency, and anemia. Among them, two focused on both maternal and child health, two were targeted at infants under 3 years, and two programs to benefit primary and middle school students. The other two programs focused on breastfeeding promotion and child obesity interventions respectively. Table 2 summarizes the target population, implementation area, content, and intervention effects of these programs.

**Table 2:** Overview of nutrition intervention programs during the MDGs

Program	Target population	Implementation area	Implementation duration (year)	Organizer	Content	Effect on nutrition improvement
Nutrition package for 6–24 months infants [24, 25]	Infants aged 6–24 months in rural areas	341 national poverty counties in 21 provinces*	Since 2012	MoH and ACWF	Providing a “nutrition package” (complementary food supplement powder, including various nutrient such us protein and micronutrients) free of charge to children aged 6–24 months per day and provision of nutrition knowledge and skills for parents on infant feeding.	• One year after the implementation of the project, the anemia rate of infants in the monitored areas dropped from 32.9 to 26.0%, stunting rate dropped from 10.1 to 8.4%, incidence of diarrhea in infants dropped from 14.2 to 9.4%, and average cost of diarrhea in infants aged 6–24 months dropped from 98.8 yuan to 74.4 yuan. • Parents’ nutrition knowledge significantly improved.
Integrated Early Childhood Development (IECD) [26]	Infants aged 0–3 years in rural areas	160 poverty villages in Shanxi and Guizhou province	Since 2013	UNICEF, PAOSC HFPC, ACWF	Providing comprehensive interventions, including nutrition supplementation, health service delivery, and child protection to infants aged 0–3 years, to explore intervention models for integrated early childhood development and child protection.	The project is still under implementation, and the results of the evaluation have not been released yet.
Improving nutrition, food safety and food security for China’s most vulnerable women and children (CFSN) [27, 28]	The high-risk population of 1.2 million children and women of childbearing age	In six of the poorest counties in western China	2009–2013	WHO, FAO, ILO, UNDP, UNESCO, UNICEF, UNIDO, and WFP, over 20 Chinese ministries at the central and local level	• Providing a basis for policy formulation by providing reliable and timely information on the scale, distribution, type, and causes of malnutrition in China. • Increasing emphasis on exclusive breastfeeding, provision of nutritional supplements, and school-based interventions to develop comprehensive programs to alleviate hunger and malnutrition among children. • Making production, processing, and production of infant and young child food safer through a regulation of shared responsibility.	• The rate of underweight and stunting for 6 to 23-month-infants decreased by 58.2% and 35.9% respectively. • The consumed proportion of micronutrient food in the pilot area increased from 21.3 to 30.1%. • Different groups such as women, children, and teachers have greatly improved their knowledge of food safety knowledge. • There was a 33.8% decrease in the prevalence of anemia and a 46% decrease in vitamin A deficiency and insufficiency in the pilot areas.
Supplementation of folic acid to prevent neural tube defects [29–31]	Women in the period of 3 months before pregnancy to the first trimester of pregnancy	Rural areas in China	Since 2009	MoH	Providing folic acid supplements to women in the period of 3 months before pregnancy to the first trimester of pregnancy for free and promotes folic acid knowledge to the gravidas and their families.	• After the implementation of this project, the incidence of neonatal neural tube defects in China decreased significantly, from 11.96/10000 in 2000 to 2.18/10000 in 2015. • The incidence of neural tube defects in China dropped from the 3rd highest incidence of birth defects to 9th.
Nutrition improvement program for rural compulsory education students [32–34]	Compulsory education students (grades 1 to 9) in rural area	Of 699 poverty counties in 21 provinces and corps^#^	Since 2011	MoE, MoF, MoH, and other 16 departments	The government provided nutritional dietary subsidies to rural compulsory education students in poverty areas, with a standard of 3 yuan per person per day (excluding weekend and holiday), which increased to 4 yuan since October 2014.	• Data monitored by the China CDC (2012–2015) showed that average height of boys and girls in 2015 was 1.2–1.4 cm higher than that of 2012, and the average weight increased by 0.7 kg and 0.8 kg, which was higher than the average growth rate of rural students in China. • The anemia rate decreased from 17.0% in 2012 to 7.8% in 2015.
Milk plan for student in China [35, 36]	Primary and middle school students in the urban area	Urban area	Since 2000	Chinese MoA, HGPC, MoE, MoF, etc.	Providing “student milk” to primary and middle school students.	• It improved the nutritional status of primary and middle school students, expanded the publicity and education of drinking milk and health, and created a good atmosphere for the development of the dairy industry. • Children’s BMI improved obviously, but the short-term height growth is small; the long-term continuous drinking of student milk has a greater effect on height.
Baby-friendly hospital [37]	Pregnant women, lactating women, and baby	Whole country	Since 1992	HFPC	In 1992, China has begun to establish baby-friendly hospitals in accordance with 10 measures to protect, promote, and support breastfeeding by the WHO and UNICEF, which aims to give full play to the important role of hospitals in promoting breastfeeding.	No nationwide data for the effects on baby-friendly hospital.
Happy ten minutes [38]	Primary students	Whole country	Since 2012	Chinese CDC and ILSI Focal Point in China	Except for the daily physical education curriculum set up in the school, teachers organize a 10-min simple and interesting exercise activity for students on each learning day.	• After the implementation of the project, the BMI-Z score of primary school students was improved.

*MoH* Ministry of Health, *ACWF* All-China Women’s Federation, *PAOSC* Poverty Alleviation Office of the State Council, *HFPC* Health and Family Planning Commission, *FAO* Food and Agriculture Organization, *ILO* International Labor Organization, *UNDP* United Nations Development Program, *UNESCO* United Nations Educational Scientific and Cultural Organization, *UNICEF* United Nations International Children’s Emergency Fund, *UNIDO* United Nations Industrial Development Organization, *WFP* World Food Program, *GAIN* Global Alliance for Improved Nutrition, *MoE* Ministry of Education, *MoF*: Ministry of Finance, *MoA* Ministry of Agriculture, *ILSI* International Life Sciences Institute

*21 provinces: Hubei, Shanxi, Inner Mongolia, Jilin, Heilongjiang, Anhui, Jiangxi, Henan, Hubei, Hunan, Guangxi, Chongqing, Sichuan, Guizhou, Yunnan, Tibet, Shaanxi, Gansu, Qinghai, Ningxia, Xinjiang

^#^21 provinces and crops: Hubei, Shanxi, Inner Mongolia, Jilin, Heilongjiang, Anhui, Jiangxi, Henan, Hubei, Hunan, Guangxi, Chongqing, Sichuan, Guizhou, Yunnan, Tibet, Shaanxi, Gansu, Qinghai, Ningxia, Xinjiang, crops

### Qualitative analysis on selected nutrition programs

Of the eight nutrition intervention programs, the *Nutrition Package for 6-24 months Infants Program* (*Nutrition Package Program*) and the *Nutrition Improvement Program for Rural Compulsory Education Students* (*Nutrition Improvement Program*) were the two most extensively implemented as well as effective nutrition interventions in China and played an essential role in the improvement of child nutrition (see Table 2 for details). Based on the qualitative interviews, we analyzed the enabling factors, challenges, and stakeholders’ views on sustainable development of the two programs.

#### The Nutrition Package for 6-24 months Infants Program

##### Enabling factors

According to the respondents, the successful implementation of the *nutrition package program* was attributed largely to (1) the efficient execution of a three-tiered network consisting of a county-township-village system, (2) cooperation between government departments and NGOs, and (3) support from national policies and funding.

**Table:** 

**Cooperation of government sectors and NGOs:** “*MoH issued documents and notices to stipulate tendering and procurement requirements, and hold annual meetings for the promotion of nutrition improvement projects.”* (informant from MoH) *“ACWF mainly undertook the work of mobilization, publicity and family education.”* (informant from ACWF)	
**Support from national policies and funding:** *“National finance allocated 100 million financial support for purchasing the nutrition packages, while many enterprises provide financial support for the promotion of nutrition packages.”* (informant from MoH)	

##### Challenges in program implementation

The qualitative analysis found four major challenges: (1) the “low price” bidding principle reduced the quality of nutrition packages, (2) funding shortages of local government, (3) nutrition packs stored in hot environments, resulting in fatty acids degradation, and (4) floating children (children migrating to urban with their rural-to-urban migrant worker parents) receive limited nutrition packages.

**Table:** 

**The “low price” bidding principle:** “*Many companies bid to make nutritional packages, the principle that local governments must choose the company with the lowest bid causing price competition reduced the quality of nutrition packages.”* (informant from CDRF)	
**Funding shortages of local government**^**#**^**:** *“The funds for publicity, stuff training, quality control and evaluation of the program, which need local budget paid in this project, is insufficient, as the local government is too poor to afford that.”* (informant from CDRF)	
**Floating children:** *“A large number of floating children who migrant to urban with their rural-to-urban migrant worker parents cannot get nutrition packages as the packages were issued according to household registration.”* (informant form CDRF)	

^#^The implementation Plan of *The Nutrition Package for 6-24 months Infants Program* stated that the national finance pays for producing nutrition package, and the local finance support funds for project publicity, stuff training, quality control, and evaluation [39].

##### Stakeholders’ views on sustainable development

In order to achieve sustainability of the programs, key informants from across departments proposed improvements in cost-effectiveness, a larger population reach, and practical strategies.

**Table:** 

**Central finance supporting for poor local government:** *“The national budget should arrange a sum of money to help some poor local government for stuff training, publicity, evaluation etc...”* (informant from MoH of Yunnan province)	
**Strengthen bidding management**: *“Scientific and technical bidding principle are needed, apart from the price, the quality of nutrition packages should also be taken into consideration.”* (officer of CCTF)	
**Strengthen evaluation:** *“Sufficient evaluations are needed for finding and solving problems and adjusting implementation plan in time.”* (professor from Wuhan University)	

#### Nutrition Improvement Program for Rural Compulsory Education Students

##### Enabling factors

Findings from the qualitative interviews indicated three main factors that were effective in promoting the implementation of the nutrition improvement program: (1) active promotion, (2) technical guidance from government, and (3) collaboration among multi-sectors.

**Table:** 

**Active promotion:** *“MoE of Yunnan province have been trying to publicize the regulations and objectives in schools, and some media has also reported the project.”* (informant from MoE of Yunnan province)	
**Technical guidance from government:** *“Chinese CDC has developed an ‘electronic nutritionist’ for guiding school canteens to conduct balanced diet, the Yunnan government also formulate recipes for the school based on Yunnan local ingredients.”* (informant from MoE of Yunnan province)	
**Collaboration between government sectors:** *“we (Chinese CDC) were responsible for the monitoring and evaluation of the program. We organized monitoring and evaluation, collected data such as height and weight, and detect hemoglobin, VA and VD every year.”* (informant from Nutrition Department of Chinese CDC) *“We (MoE) were mainly responsible for safety of food and funds*.*”* (informant from MoE)	

##### Challenges in program implementation

Since the project covered a wide geographical area, involved a large number of departments, and had no precedent to follow, it has faced several problems: (1) misconceptions from parents, (2) shortage of operational funding, (3) lack of nutrition professionals, and (4) poor quality of monitoring. Additionally, the program has excluded preschool children and poor students in the urban cities, which caused unfairness and inequities.

**Table:** 

**Misconceptions from parents:** *“Some parents simply regarded the program as free lunch, causing ‘crowding-out effects’ in many regions.”* (informant from MoE)	
**Shortage of operational funding*:*** *“The national finances did not allocate the services and work expenses, which need to be supported by the local authorities, so that the project monitoring, personnel training work is difficult to carry out due to the shortage of local finance.”* (informant from MoE of Yunnan province)*.*	
**Lack of nutrition professionals:** *“The canteen staff knew very little about nutrition knowledge, making it hard to provide students with a balanced meal.”* (informant from Nutrition Department of Chinese CDC)	
**Poor monitoring:** *“The monitoring sample size is too large, work fund and manpower cannot meet the needs of the monitoring work, staff lacks regular physical examination and questionnaire survey training, which make it is difficult to guarantee the quality of monitoring data.”* (informant from Hubei CDC)	
**Unfairness and inequities:** “*In fact, children in preschool, was not covered by the program.”* (informant from Peking University) *“Student participating in the program is based on household registration, only rural students could be included, without taking into account the urban poor students.”* (Committee of experts on the *Nutrition Improvement Program*)	

##### Stakeholders’ views on sustainable development

In response to these challenges, informants from various sectors (Nutrition Department of Chinese CDC, UNICEF, CDRF, Hubei CDC, MoE of Hubei and Kunming Medical University) put forward recommendations for sustainability of the project as presented in the following table.

**Table:** 

**Reconsider the inclusion criteria of target children:** *“The inclusion criteria should be changed, for example, to include children from urban poor families. Rural household registration should not be used as a standard of entry.”* (Committee of experts on the *Nutrition Improvement Program*)	
**Pay attention to child obesity:** *“At present, undernutrition is not completely solved, while the problem of overweight and obesity has emerged. In this case, the project should pay attention to both undernutrition and overweight and obese children.”* (informant from CDRF)	
**Develop adequate legislation:** *“The government could introduce a law on nutritional protection for students, and establish a sound nutritional feeding system.”* (Director from MoE of Hubei)	
**Enhance the nutrition knowledge and skill of students, teachers, parents and canteen staff:** *“Promoting nutrition and health education of students, teachers and parents, to cultivate good diet habits for students.”* (informant from Child & adolescent nutrition, UNICEF) *“It is important to train the canteen staff to improve their nutritional knowledge and catering skills.”* (informant from Nutrition Department of Chinese CDC)	
**Scientific evaluation: “***Current evaluation indicators of height and weight are not only related to diet, but also affected by other factors including sleep, exercise, hormones and many other factors. So, this monitoring design should be more reasonable.”* (Committee of experts on the *Nutrition Improvement Program*)	

## Discussion

This study analyzed the main content of nutrition-related policies, successes, and challenges and lessons learned from the leading national nutrition intervention projects to understand the experiences of maternal and child nutrition strategies in China that could help achieve the nutrition-related 2030 SDGs in China.

### Implementation of nutrition-related policies

During the MDGs, China promulgated six nutrition policies and regulations to promote nutrition and health conditions of Chinese woman and children. These nutrition policies focused on the nutritional needs of women and children at certain age and economic conditions. For example, breastfeeding and complementary feeding improvement of infants and the nutrition of school children from poor rural families. Additionally, the nutritional policies put forward specific nutritional goals to be achieved in certain years and identified and prioritized key areas and populations, as well as relevant technical guidance for nutrition improvement.

While progress was made, we also found a number of shortcomings of nutrition policies. First, the issued policies put most of the focus on undernutrition, complementary feeding, and breastfeeding improvement, while goals and guidance for prevention of overweight and obesity were rare. Secondly, currently, there are no laws to guarantee the effective implementation of relevant policies throughout provinces, cities, and regions in China. For example, the *China Nutrition Improvement Action Plan* issued by the State Council in 1997 has not been implemented widely until 2003 [40]. It is not possible to clarify the responsibilities of various departments and guarantee the cultivation and maintenance of nutrition professionals. In contrast, Japan and the USA have made major breakthroughs in nutrition improvement programs. The USA enacted a series of federal nutrition-related laws in the 1960s and 1970s to reduce hunger and to guarantee implementation, such as the *National School Nutrition Lunch Act* [41], and in Japan the *school feeding law*, *nutrition improvement law*, and *Nutritionist Law* [42].

### Experiences of nutrition programs and implications for achieving the Sustainable Development Goals

China implemented eight nationwide maternal and child nutrition intervention programs during the MDGs period. Several government sectors and NGOs were involved in developing and implementing each of the programs, for example, MoH, collaborated with MoF for *Nutrition Package Program*, and MoE, MoF, MoH, and CDC worked collaboratively together for the *Nutrition Improvement Program*. Multi-sectoral collaboration among government sectors including health, finance, and education sectors built a comprehensive implementation system for nutrition interventions. The collaboration between health-related NGOs including UNICEF, ACWF, CDRF, and WHO who had international experience and innovative ideas was one of strongest enabling factors for maternal and child nutrition improvement interventions in China. Additionally, we found that China prioritized and focused heavily on improvements to reduce child undernutrition.

For reducing undernutrition, six programs have been launched by the Chinese government and NGOs, achieving positive results. These interventions have reduced the incidence and prevalence of maternal and child undernutrition including anemia, stunting, underweight, and neural tube defects (Table 2). But more progress needs to be made in undernutrition interventions, for instance, the target population has been mainly infants aged 6–36 months and primary and middle school students, with less attention on preschool children 36–48 months of age. Further, as shown in the qualitative analysis, the two key nutrition interventions in China continue to face challenges such as insufficient local operational funds and lack of monitoring. There are four SDGs related to undernutrition, including low birth weight, stunting and wasting in in children younger than 5 years, and anemia in reproductive-age women. In China, the low birth weight rate was 3.3% in 2013, in a low level [12]. The wasting rate of children under 5 years was 2.7%, which has already reached the SDG target in 2016 [43]. For child stunting and anemia in women, policies and intervention programs have achieved positive results (Table 2). The stunting prevalence of children under 5 years from 2000 to 2016 has achieved 41% reduction [43] and 70% and 40% reduction for anemia in lactating women and pregnant women from 2002 to 2013, respectively [12]. Therefore, the SDG nutrition targets of child stunting, low birth rate, and anemia in women in China could likely be achieved if the challenges mentioned above could be addressed with multi-sector approaches.

For EBF improvements, we found although several policies and guidance for EBF improvement were issued, they have not been implemented well. The EBF rate for infants aged 6 months in China in 2013 was only 20.8% [12], which is far away from the SDG goal of 50% [10, 11]. On one hand, intervention actions on EBF improvement were few. The baby-friendly hospital is the only nationwide intervention program in China. On the other hand, although China started to establish baby-friendly hospitals (BFHI) in 1992, and had a large number (6711) of BFHI [44], few evaluations have been conducted on its impact for EBF improvement. In our literature search, we only found two hospital-based studies [45, 46] and one survey [47] evaluating the effects improvement on breastfeeding rates, and no nationwide evaluations have been done. Sound monitoring and evaluation system has not been established to evaluate the effectiveness of BFHI, which may be one of the reasons why the EBF rate of infants aged 6 months in China have been low, despite the large number of hospitals participating and that the program was established in the early 1990s. Studies have shown that education and consults in communities are effective actions for EBF improvement [48]. Comprehensive evaluations of BFHI, and more targeted interventions combined with government regulations and NGO support, including community and field technical assistance should be initiated in China to achieve the EBF SDG goal.

Regarding overweight and obesity, although China’s childhood overweight and obesity rates were already at a high level (the prevalence of overweight for children aged 1–4 years in 2016 was 11.9% and 6.9% for obesity [43]), few regulation and targets have been issued to prevent and control obesity, and nationwide intervention programs on prevention or treatment of child obesity are rare. The school-based program “*Happy ten minutes*” is the only nationwide intervention on child obesity. Furthermore, this program only targeted at primary students, without obesity intervention actions for children in pre- and middle school. From 2000 to 2016, the rate of overweight and obesity of children under 1–4 years has been increasing, from 9.5% and 3.9% to 11.9% and 6.9% respectively [43]; the SDG of no increase in childhood overweight has not been achieved. Therefore, more coordinated national overweight and obesity polices and intervention actions for pre- and middle school children are urgently needed in order for China to achieve the SDG goal of no increase in childhood overweight. Optimal nutrition is related to many factors, such as appropriate complementary feeding, lack of infectious diseases, access to health care and a nutritious and affordable food supply, and a healthy environment, all of which also should be considered as priorities by government to achieve the SDGs.

### Lessons learned from China for other developing countries

China’s positive experience on reducing undernutrition provides several important lessons for other developing countries with high rates of undernutrition: (1) achieving rapid reductions in undernutrition clearly requires high-level political attention. The Chinese government has realized the importance of nutrition, especially for children and women. During the MDGs era, a large number of nutrition policies were promulgated, not only at the national level, but also poverty-stricken and rural areas. These nutrition policies are the basis for nutrition work. (2) Policies with priority targets are another reason for the progress and achievement made in China through the nutrition interventions. Prioritized nutrition, especially in poverty and remote areas, and the focus on 0–24-month-old children and women are important for improving nutrition. Further more specific nutritional goals and technical guidance for nutrition policy are also essential. (3) Nutrition interventions play an essential role in improving maternal and child nutrition and require multi-sectoral collaboration of related government sectors and NGOs and a comprehensive monitoring system. The implementation of nutrition intervention in China involves multiple sectors, such as finance, health, and education; only by clarifying their respective responsibilities and cooperating with each other can the programs be implemented successfully. The NGOs such as UNICEF and ACWF are important collaborators in conducting nutrition intervention pilots, disseminating nutrition knowledge, and monitoring intervention effects. Governments need to establish public-private partnerships with NGOs. A monitoring system is essential for nutrition interventions, not only to evaluate trends and progress, but also to help identify gaps, problems, and any unintended consequences. In addition, the challenges that China is facing, such as lack of local budgets, problems with monitoring systems, lack of a nutrition workforce, and large number of floating children, may alert other developing countries that when planning and implementation nutrition interventions, more attention needs to be paid to minimize and avoid similar problems.

There were several potential limitations that should be acknowledged in this study. Although we conducted a comprehensive review of maternal and child nutrition policies implemented during the MDGs era in China, we found the evaluation of policies are insufficient, and therefore, we cannot adequately assess effectiveness or impact. Also, we only assessed the effects of nutrition intervention policies on nutrition improvement and evaluation of implementation and cost-effectiveness of the two projects is lacking. Our qualitative interviews were only for project leaders and managers. We did not interview the target populations of those two projects. Still, our findings will be beneficial in informing efforts to achieve the SDG nutrition goals in China and provide lessons learned for other developing countries in reducing the prevalence of malnutrition.

## Conclusion

Our findings document that the nutrition policies and nutrition intervention programs the Chinese government promulgated during the MDGs period have improved maternal and child nutrition, especially undernutrition in children. Collectively, the nutrition policies have created a strong supportive environment for nutrition improvement in China; however, policies have put the main focus on reducing undernourished children and women and improving breastfeeding, with limited attention on overweight. Overall, China has made major contributions to the rapid decline of undernutrition and is likely with continued efforts to achieve the SDGs of child wasting, stunting, low birth weight, and anemia in reproductive-age women. However, appropriate policies and interventions are needed to improve breastfeeding rates and to control and prevent childhood obesity in years to come.

## Supplementary Information

**Additional file 1: Table S1.** Information sheet of interview participants.

## Data Availability

The datasets used and/or analyzed during the current study are available from the corresponding author on reasonable request.

## Abbreviations

MDGs: The Millennium Development Goals
UN: United Nations
WHO: World Health Organization
EBF: Exclusive breastfeeding
SGDs: Sustainable Development Goals
NGOs: Non-governmental organizations
CNKI: China National Knowledge Infrastructure database
MoE: Ministry of Education
HFPC: National Health and Family Planning Commission of China
UNICEF: United Nations International Children’s Fund
CDCs: Centers for Disease Control
CCTF: China Children and Teenager’s Fund
CDRF: China Development Research Foundation
ACWF: All-China Women’s Federation
PAOSC: Poverty Alleviation Office of the State Council
FAO: Food and Agriculture Organization
ILO: International Labor Organization
UNDP: United Nations Development Program
UNESCO: United Nations Educational Scientific and Cultural Organization
UNIDO: United Nations Industrial Development Organization
WFP: World Food Program
GAIN: Global Alliance for Improved Nutrition
MoA: Ministry of Agriculture
ILSI: International Life Sciences Institute
BFHI: Baby-friendly hospital
