# Supplementary material for: Improving maternal and child nutrition in China: an analysis of nutrition policies and programs initiated during the 2000–2015 Millennium Development Goals era and implications for achieving the Sustainable Development Goals
Source: J Health Popul Nutr. 2020 Dec 2;39:12. doi: 10.1186/s41043-020-00221-y (PMC7709233; doi:10.1186/s41043-020-00221-y)
Supplement: Supplementary file 1 — Additional file 1: Table S1. Information sheet of interview participants. [file 41043_2020_221_MOESM1_ESM.doc]

Table S1. Information sheet of interview participants

|  | **Gender** | **Department** | **Specialities** | **Titles** |
| --- | --- | --- | --- | --- |
| **Beijing Municipality** | | | | |
| 1 | F | UNICEF | Child & adolescent nutrition | Director of department |
| 2 | F | Children and family department of All-China Women's Federation | Family education | Vice director of department |
| 3 | F | China Children and Teenagers’ Fund | Child nutrition package | - |
| 4 | F | Maternal and Child Health Department of Chinese CDC | Child nutrition | - |
| 5 | M | Nutrition department of Public Health School, Peking University | Nutrition and food, child nutrition | Director |
| 6 | F | China Development Research Foundation, Children section | Nutrition Improvement Program for Rural Compulsory Education Students | Vice director |
| 7 | F | China Development Research Foundation, Children sector |  | - |
| 8 | F | Maternal Health Sector of Maternal and Child Health Department, MoH | Maternal health | Director, professor |
| 9 | F | Student’s Nutrition Office, Nutrition Department of Chinese CDC | Student office | Vice director |
| 10 | F | Maternal Health Sector of Maternal and Child Health Department, MoH | Child nutrition and health | Director |
| **Hubei Province** | | | | |
| 1 | F | Child health department, Maternal and child hospital in Hubei | Child health | Vice director |
| 2 | F | School of public health, Wuhan University | Maternal and child health | Professor |
| 3 | F | Institute of Health Surveillance and Prevention, Hubei CDC | Nutrition | Director |
| 4 | M | Institute of Health Surveillance and Prevention, Hubei CDC | Nutrition | - |
| 5 | M | Maternal and Child Health Sector, MoH of Hubei | Child health | - |
| 6 | M | Department of Physical, Health and Arts, MoE of Hubei | Student education | Director |
| **Yunnan Province** | | | | |
| 1 | M | Maternal and Child Health Sector, MoH of Yunnan | Child Nutrition Package | - |
| 2 | F | School of public health, Kunming medical university | Students nutrition, school meal | Professor |
| 3 | M | Financial department, MoE of Yunnan | School meal program | Director |
| 4 | M | Child health department, Maternal and child hospital in Yunnan | Child health | Vice Director |
| 5 | M | School of public health, Kunming medical university | Nutrition | Director, professor |
| 6 | M | Institute of School Health department, Yunnan CDC | Student health | Director |
| 7 | M | Institute of Nutrition and Food health, Yunnan CDC | Nutrition | Director |
